# The numerology of gender: gendered perceptions of even and odd numbers

**DOI:** 10.3389/fpsyg.2015.00810

**Published:** 2015-06-11

**Authors:** James E. B. Wilkie, Galen V. Bodenhausen

**Affiliations:** ^1^Department of Marketing, University of Notre DameNotre Dame, IN, USA; ^2^Departments of Psychology and Marketing, Northwestern UniversityEvanston, IL, USA

**Keywords:** numbers, gender, implicit associations, sex differences, social stereotypes

## Abstract

Do numbers have gender? Wilkie and Bodenhausen (2012) examined this issue in a series of experiments on perceived gender. They examined the perceived gender of baby faces and foreign names. Arbitrary numbers presented with these faces and names influenced their perceived gender. Specifically, odd numbers connoted masculinity, while even numbers connoted femininity. In two new studies (total *N* = 315), we further examined the gendering of numbers. The first study examined explicit ratings of 1-digit numbers. We confirmed that odd numbers seemed masculine while even numbers seemed feminine. Although both men and women showed this pattern, it was more pronounced among women. We also examined whether this pattern holds for automatic as well as deliberated reactions. Results of an Implicit Association Test showed that it did, but only among the women. The implicit and explicit patterns of numerical gender ascription were moderately correlated. The second study examined explicit perceptions of 2-digit numbers. Again, women viewed odd numbers as more masculine and less feminine than even numbers. However, men viewed 2-digit numbers as relatively masculine, regardless of whether they were even or odd. These results indicate that women and men impute gender to numbers in different ways and to different extents. We discuss possible implications for understanding how people relate to and are influenced by numbers in a variety of real-life contexts.

## Introduction

Gendered information plays a prominent role in how people interpret both the physical and social environments in which they live. Research has shown that individuals begin to acquire information about gender as early as 6 months of age, when infants start to distinguish males and females (see Martin et al., 2002). From this basic foundation, social conditioning processes (Bussey and Bandura, 1999), experienced gender differences (Cejka and Eagly, 1999), and gendered language structures (Boroditsky et al., 2003) provide avenues for learning and reinforcement regarding which objects and actions are considered to be masculine and which are feminine. In this way, representations of much of the social world come to be imbued with gender connotations to one degree or another.

Although it is well known that concrete cultural artifacts like toys and clothing can have strong gender associations, recent research has suggested that gendered thinking extends even into the realm of very abstract and seemingly asocial concepts. For instance, Wilkie and Bodenhausen (2012) found that participants rated the concept “even numbers” as relatively feminine and the concept of “odd numbers” as relatively masculine. Further, they found that when odd numbers were arbitrarily paired with gender-ambiguous stimuli (baby faces or foreign names), the stimuli were rated as more likely to be male, yet when even numbers accompanied the same stimuli, they were more likely to be seen as female.

The ascription of gender to objects, actions, and concepts can be highly consequential, because such information is commonly used to guide people’s judgments and decision making. Males and females are expected to behave in accordance with established gender roles and can be penalized psychologically (e.g., Crocker and Major, 1989; Pleck et al., 1993; Crocker et al., 1998; Major and O’Brien, 2005) and economically (Crocker et al., 1998; Rudman, 1998; Rudman and Glick, 1999, 2001) for violations of culturally established gender norms. Research suggests that these pressures result in people having to devote cognitive resources in efforts to monitor and maintain either a relatively masculine or feminine gender identity. Research has also suggested that there are gender differences in how much these pressures seem to affect behavior, as males (in the U.S.) tend to face greater penalties for gender transgressions than females do. For instance, Gal and Wilkie (2010) found that American males, but not females, were more likely to choose gender-conforming products (i.e., food and household items) when they had sufficient cognitive resources available to consider the consequences of their choices. Thus, in order to predict evaluations and behavior in a choice context, it is important to consider the gender connotations of available options as well as the sex of the individual making the decision.

Numbers are abstract concepts, and numerical parity (i.e., whether a number is even or odd) is central to their mental representation (Shepard et al., 1975). Developmental research indicates that parity becomes an integral part of number representation from about the 4th grade onward (Berch et al., 1999). Thus, if parity is indeed inherently linked to gender, then gender is likely to be a pervasive component of number representation. The present research sought to expand upon the prior studies of Wilkie and Bodenhausen (2012) by directly documenting the extent to which specific numbers are gendered and to explore the possibility of sex differences in numerical gendering. Secondarily, we also examined whether there are differences in how much people like even vs. odd numbers and whether such differences are related to numerical gender connotations. Prior research suggests that people may have the tendency to prefer even numbers over odd numbers because mathematical operations involving even numbers are typically experienced as less difficult than ones involving odd numbers (Knight and Behrens, 1928; Hines, 1990). When learning basic multiplication, the correct answer is an even number 75% of the time on average [because whenever an even number is multiplied with another number, its product must be even regardless of whether the multiplier is odd or even], which creates greater familiarity with even numbers (Lochy et al., 2000). Related to this finding, previous research has also found that people tend to process even numbers more rapidly and fluently than odd ones (e.g., Hines, 1990), and much research has documented that processing fluency elicits positive affect toward salient stimuli (see Winkielman et al., 2003). Furthermore, the gendering of numbers might also imply differential liking of even and odd numbers. Eagly and Mladinic (1994) noted that femininity stereotypes (e.g., warm, nurturing, emotionally sensitive) imply likableness much more than masculinity stereotypes (e.g., strong, independent, competitive), a phenomenon Eagly and Mladinic dubbed the “women-are-wonderful” effect. Thus, even numbers may seem more likable than odd numbers by virtue of their greater perceived femininity. This possibility was explored in the present studies, as well as the question of whether such numerical connotations exist to a similar extent among men and women.

Thus, the present experiments examined: (1) whether people associate specific even and odd numbers with a particular gender, (2) whether there is an affective preference for even numbers over odd numbers, and (3) whether participant sex moderates either or both of these phenomena. Because the participants in Wilkie and Bodenhausen’s (2012) experiments were primarily women, the possibility of sex differences could not be explored in those earlier studies. These questions were examined using both implicit and explicit measures. More specifically, in Study 1, we used the Implicit Association Test (IAT; Greenwald et al., 1998) to examine the automatic gender connotations of specific numbers from 1 to 99. The IAT is a widely used technique for measuring the strength of respondents’ tendency to mentally associate particular concepts with one another. In addition, at the explicit level, we examined the gender connotations and likableness of single-digit numbers (Study 1) and double-digit numbers (Study 2).

## Study 1

Study 1 examined both implicit and explicit ascription of gender to numerals. We specifically tested the prediction that the odd digits would be associated with masculinity whereas even digits would be associated with femininity. Predictions regarding the number 0 were less straightforward than predictions regarding positive integers. Although multi-digit numbers ending in 0 are clearly even numbers, the number 0 itself is an interesting case. To ascertain whether lay people readily categorize 0 as an even number, we conducted a pretest with 203 respondents on Amazon Mechanical Turk, in which the participants were simply asked whether they considered 0 to be an even number, an odd number, or neither even nor odd. Only a minority of respondents said they considered 0 to be an even number (39%), while the majority regarded 0 as neither even nor odd (59%) and a few (2%) categorized it as an odd number. Given this pattern, we hypothesized that 0 would not be judged in the same way as other even numbers.

### Method

#### Participants and Design

We recruited the largest student sample we could obtain within a single academic term, which consisted of 119 undergraduates who received extra credit in return for their participation. The sample consisted of 75 women, 39 men, and 5 individuals who did not specify their sex; it was 67% White and ranged in age from 18 to 40 (*M* = 20.44, SD = 2.62). Participants responded to both odd and even numbers, and participant sex was the only between-participants variable. The research was approved by the Institutional Review Board of the University of Notre Dame, and informed consent was obtained from all participants.

#### Materials and Procedure

Participants reported to a lab, where they received general instructions from a female experimenter and then completed a series of tasks on a computer. First, they completed a measure of automatic number-gender associations. Next, they provided explicit ratings of the gender connotations of both even and odd individual digits. Finally, they provided basic demographic information.

As a measure of automatic number-gender associations, we used the IAT. In contrast to explicit measures, an advantage of the IAT is that it reveals the influence of spontaneous mental associations, regardless of whether or not people have accurate self-insight into the existence of these associations. Thus, its use allows us to test for numeric gender associations that people may hold without their awareness.

The IAT involves the rapid categorization of stimuli into four different categories: two concepts related to the target objects (here, even and odd numbers) and two attribute categories (here, feminine and masculine traits). When numbers appeared on the screen, participants were required to categorize them as even or odd. When trait words appeared on the screen, participants were required to categorize them as feminine or masculine. The rule governing which response key to use was signaled by category labels placed in the upper left and upper right corners of the screen. In critical trial blocks, words, and numbers were intermixed, and the response rule for both attribute and target discrimination was simultaneously presented on the screen. For example, when “Odd Numbers” and “Feminine Traits” categories appeared on the upper left side of the screen and “Even Numbers” and “Masculine Traits” appeared on the upper right, participants were required to categorize odd numbers and feminine traits by pressing the “E” key on the left side of the keyboard and to categorize even numbers and masculine traits by pressing the “I” key on the right side. When an error was made, an “X” appeared on the screen until participants chose the correct response.

The even numbers were randomly drawn for each participant from a bin that contained all even numbers that ranged from 2 to 98, and the odd stimuli were randomly sampled from all the odd numbers from 1 to 99. For the attribute concepts, stimuli consisted of five words that corresponded to stereotypically masculine/agentic traits (i.e., “Brave,” “Strong,” “Manly,” “Assertive,” and “Aggressive”) and five words corresponding to stereotypically feminine/communal traits (i.e., “Nurturing,” “Empathetic,” “Girly,” “Gentle,” and “Soft”); for an overview of the agentic and communal content of gender stereotypes, see Abele (2003). Participants were instructed to categorize stimuli as quickly and accurately as possible by pressing the appropriate response key.

The study followed standard IAT procedures (Greenwald et al., 1998) and was administered via Millisecond’s Inquisit software. The IAT involved seven blocks of trials. Blocks 1, 2, and 5 were practice blocks in which the participants practiced the simple task of categorizing either the number stimuli or the masculine and feminine traits. In the remaining blocks (i.e., Blocks 3, 4, 6, and 7), participants simultaneously categorized both sets of stimuli. For approximately half of the participants, Blocks 3 and 4 consisted of trials where even numbers and feminine words (and odd numbers and masculine traits) shared the same response, and the response rule switched to pairings of even/masculine and odd/feminine for Blocks 6 and 7. For the rest of the participants, this order was reversed.

Following the IAT, participants completed explicit ratings of specific 1-digit numbers. Although masculinity–femininity is often perceived as a single bipolar dimension, past research has indicated that masculinity and femininity can coexist (e.g., Bem, 1974; Heilbrun, 1976); for this reason we collected separate unipolar measures of perceived masculinity and femininity. We used two strategies to explicitly measure numerical gender. First, we asked participants to directly rate each number in terms of its masculinity and femininity. Second, we asked them to rate each number in terms of specific gendered traits. Masculinity is associated with agentic traits (i.e., traits reflecting the capacity or tendency for autonomous action and the exertion of power), while femininity is associated with communal traits (i.e., traits reflecting nurturance and a warm interpersonal orientation; see Abele, 2003). Thus, we had participants rate the numbers on two masculine-agentic traits (*independent* and *strong*) and two feminine–communal traits (*friendly* and *soft*). All of the respondents completed all of these gender measures for each of the nine positive single-digit numbers, as well as 0. They rated a given number on each of the rating scales before moving on to the next number. Finally, we also collected ratings of the verbal concepts “odd numbers” and “even numbers” on the same rating scales (e.g., “How masculine are odd numbers?”).

We assessed liking for the individual digits by having respondents rate each one in terms of its positivity, likableness, and pleasantness. All of the collected ratings were made on response scales ranging from 1 (“not at all”) to 7 (“extremely”).

### Results

#### IAT Scoring

In the IAT, strength of association is assessed by comparing participants’ reaction times to different attribute–target pairings, with faster reaction times interpreted as a stronger association. For example, people who associate even numbers with femininity and odd numbers with masculinity should be faster to categorize stimuli when the category pairings are even numbers/feminine traits and odd numbers/masculine traits, compared to when the category pairings are the opposite.

Participants’ IAT responses were scored according to Greenwald et al.’s (2003) improved scoring algorithm in order to create a *D*-score for each participant. The *D*-score is an effect size estimate that is created by dividing differences between the mean response latencies of the two types of double-categorization blocks by the SD of all latencies in the blocks. The direction and size of the *D*-score reflects the relative strength of associations between the target concepts and attributes. Here, positive scores indicate that participants were quicker to categorize stimuli in the even numbers – feminine traits/odd numbers- masculine traits blocks than in the even numbers – masculine traits/odd numbers – feminine traits blocks. The strength of the IAT effect corresponds to the conventional criteria used to label small (0.20), moderate (0.50), and large (0.80) effects sizes of Cohen’s *d* measure. The overall mean observed *D*-score was 0.22 (SD = 0.53), which was significantly different from 0, *t*(118) = 4.47, *p* < 0.001, 95% CI [0.12,0.31]. Thus, at the implicit level, there is evidence for a numerical gender association such that even numerals are associated with femininity and odd numerals are associated with masculinity, although the overall effect is small in magnitude. It is unclear, however, if individuals are aware of such associations and, if so, whether they would express them in explicit judgments about numbers. To examine this, we next turn to the explicit ratings of the digits 0 through 9.

#### Ratings of Individual Digits

First we examined the ratings of each individual digit from 0 to 9. Results are presented in **Table 1**. Direct ratings of masculinity and femininity are presented on the left side of the table. As expected, the number 0 connoted relatively low levels of both masculinity and femininity, with no reliable difference between these two ratings. Zero does not appear to be gendered. However, the digits 1, 3, 5, 7, and 9 all showed a robust pattern of connoting more masculinity than femininity. With respect to the even digits, ratings of 2, 4, 6, and 8 showed the expected pattern of greater femininity than masculinity. We next examined the ratings of agentic and communal associations with the individual digits. To do so, for each number we combined the two agentic trait ratings (independent and strong) into an agentic score and the two communal trait ratings (friendly and soft) into a communal score. Results are presented in the right side of **Table 1**, and they follow a pattern very similar to the direct ratings of masculinity and femininity. Specifically, zero was rated low on both agentic and communal qualities, and all of the odd digits showed a pattern of being more agentic than communal while all of the even digits showed a pattern of being more communal than agentic. These directional differences were statistically reliable except in the cases of the digits 3, 4, and 8, which were only marginally significant (*p*s < 0.08). Overall, the results converged on a very consistent pattern in which odd numbers were perceived to be more masculine-agentic than even ones, while even numbers were perceived to be more feminine–communal than odd ones.

**Table 1:** Study 1 ratings of individual digits.

	Direct ratings				Trait ratings			
Number	Masculine	Feminine	Difference [95% CI]	*g_rm_*	Agency	Communion	Difference [95% CI]	*g_rm_*
**0**	3.88 (1.24)	4.01 (1.23)	-0.13 [-0.40, 0.14]	0.10	3.97 (1.37)	4.05 (1.33)	-0.08 [-0.38, 0.22]	0.06
**1**	4.87 (1.37)	3.75 (1.16)	1.11 [0.79, 1.43]	0.87	5.03 (1.44)	3.81 (1.21)	1.23 [0.90, 1.55]	0.92
**2**	3.96 (1.11)	4.70 (1.07)	-0.74 [-0.99, -0.49]	0.68	4.06 (1.20)	4.75 (1.09)	-0.69 [-0.92, -0.47]	0.60
**3**	4.30 (0.98)	3.98 (1.10)	0.31 [0.07, 0.55]	0.30	4.27 (1.14)	4.06 (1.16)	0.22 [-0.21, 0.45]	0.19
**4**	4.03 (1.07)	4.34 (1.00)	-0.31 [-0.55, -0.08]	0.30	4.13 (1.21)	4.36 (1.02)	-0.23 [-0.47, 0.01]	0.21
**5**	4.64 (1.04)	3.85 (0.93)	0.79 [0.53, 1.06]	0.80	4.74 (1.09)	3.93 (1.09)	0.81 [0.54, 1.08]	0.70
**6**	4.06 (0.96)	4.44 (0.92)	-0.38 [-0.62, -0.13]	0.40	4.19 (1.04)	4.45 (1.01)	-0.26 [-0.51, -0.01]	0.25
**7**	4.69 (1.09)	4.04 (1.06)	0.65 [0.41, 0.90]	0.60	4.83 (1.20)	4.03 (1.18)	0.80 [0.54, 1.05]	0.67
**8**	4.24 (1.03)	4.60 (1.02)	-0.35 [-0.62, -0.09]	0.34	4.40 (1.13)	4.65 (1.09)	-0.25 [-0.51, 0.01]	0.23
**9**	4.56 (1.14)	3.95 (1.10)	0.60 [0.34, 0.87]	0.54	4.65 (1.25)	3.98 (1.15)	0.67 [0.40, 0.93]	0.55

*Mean ratings of the masculinity and femininity (left columns) and agentic and communal traits (right columns) of each integer between 0 and 9. For each difference, Hedges’s g_rm_ provides a standardized effect size*.

**FIGURE 1:**
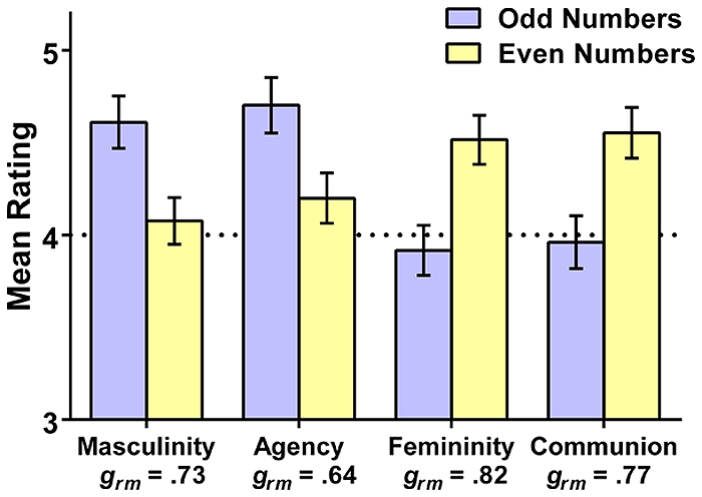
**Mean composite ratings (and 95% CIs) of the masculine, agentic, feminine, and communal qualities of the odd (1, 3, 5, 7, 9), and even (2, 4, 6, 8) digits in Study 1**. Standardized effect sizes (Hedges’s *g*_rm_) are provided for each rating type; the dotted line reflects the rating scale midpoint.

To examine the overall tendency to associate gender with odd vs. even numbers, we next compared the averaged ratings of the odd digits (i.e., 1, 3, 5, 7, and 9) and the even digits (i.e., 2, 4, 6, and 8). One participant’s results could not be included in this analysis due to missing data. Results are depicted in **Figure 1**. The pattern clearly reflects the hypothesized gendering of numbers. Odd numbers were rated as more masculine and more agentic than even numbers, whereas even numbers were rated as more feminine and more communal than odd numbers (all *t*s > 5.26, all *p*s < 0.001). The standardized effect sizes (calculated as recommended in Lakens, 2013), provided in the figure, were all in the moderate to large range.

#### Ratings of the Verbal Concepts

Participants also rated the verbal concepts “odd numbers” and “even numbers” in terms of their masculinity, agency, femininity, and communion. The concept “odd numbers” was rated as more masculine than “even numbers” [*M* (SD) = 4.86 (1.07) vs. 4.13 (1.01), respectively], Hedges’s *g*_rm_ = 0.70, and more agentic than them [*M* (SD) = 4.89 (1.16) vs. 4.26 (1.13), respectively], Hedges’s *g*_rm_ = 0.56; in contrast to “odd numbers,” the concept of “even numbers” was rated as more feminine [*M* (SD) = 4.89 (1.10) vs. 3.65 (1.26)], Hedges’s *g*_rm_ = 1.04, and more communal [*M* (SD) = 4.97 vs. 3.69], Hedges’s *g*_rm_ = 1.05. For these comparisons, all *t*s > 3.74, all *p*s < 0.001. Overall, this pattern documents moderate to large differences in the gender connotations of the general concepts of odd vs. even numbers and directly corroborates the findings from the ratings of specific numerical digits.

#### General Evaluation of Numbers

We examined whether there was a tendency to view even numbers more favorably than odd numbers, given the evidence that feminine qualities are generally perceived in more favorable ways than masculine qualities, as well as the evidence that even numbers tend to be associated with more fluent numerical processing. Participants rated each individual digit in terms of its positivity, pleasantness, and likableness. Because they were highly correlated, these three ratings were averaged for each digit to construct an overall index of evaluation. Then, we computed composite liking scores for the odd (1, 3, 5, 7, 9) and the even (2, 4, 6, 8) digits. Consistent with predictions, the composite positivity of even numbers (*M* = 4.77, SD = 0.80) was greater than that of odd numbers (*M* = 4.50, SD = 0.84), *t*(117) = 2.79, *p* = 0.006, Hedges’s *g*_rm_ = 0.33. When rating the general concepts of “odd numbers” and “even numbers” rather than specific numerical stimuli, participants again rated even numbers as higher in composite positivity than odd numbers [*M* (SD) = 5.17 (.97) vs. 4.01 (1.19), respectively], *t*(117) = 7.39, *p* < 0.001, Hedges’s *g*_rm_ = 1.07.

Correlational analyses provided evidence for a connection between perceived femininity and greater liking of numbers. The composite liking measure for the even digits was substantially correlated with perceptions of their femininity, *r* = 0.55, *p* < 0.001, 95% CI [0.42,0.67], but not their masculinity, *r* = -0.01, *p* = 0.90, 95% CI [-0.19,0.17]. The composite liking of the odd digits was also correlated with the degree to which these numbers were perceived to be feminine, *r* = 0.29, *p* < 0.001, 95% CI [0.12,0.45], but it was correlated as well with the degree to which odd numbers were perceived to be masculine, *r* = 0.33, *p* < 0.001, 95% CI [0.16,0.48].

#### Implicit/Explicit Relations

Next we examined the degree of convergence between implicit and explicit measures of numerical gendering. For these analyses, we could not use the data from 15 participants who failed to enter an identification code that was needed to connect their IAT responses (which were collected in a different computer program) to their explicit ratings. To construct an explicit measure of numerical gendering that is directly analogous to the IAT measure, which involves an inherent contrast between the associations of even vs. odd numbers, we constructed two composite scores reflecting the strength of the overall numerical gender contrast. The first contrast was based on the direct ratings of masculinity and femininity: (composite masculinity ratings of odd numbers + composite femininity ratings of even numbers) – (composite masculinity ratings of even numbers + composite femininity ratings of odd numbers). The second composite involved the same contrast pattern but substituted the agentic trait ratings for masculinity and the communal trait ratings for femininity. Larger values on these contrast measures reflect a stronger overall tendency to explicitly associate masculinity with odd numbers and femininity with even numbers. The explicit masculinity/femininity contrast was significantly correlated with IAT scores, *r* = 0.35, *p* < 0.001, 95% CI [0.17,0.51] as was the explicit agency/communion contrast, *r* = 0.34, *p* < 0.001, 95% CI [0.16,0.50]. Thus, there was a significant correspondence between the implicit and explicit gendering of numbers; people who tended to automatically associate odd numbers with masculinity and even numbers with femininity also tended to explicitly rate the numbers in this manner.

Implicit Association Test scores were also significantly correlated with the composite measure of the likableness of even numbers, *r* = 0.34, *p* < 0.001, 95% CI [0.16,0.50], but not with the likableness of odd numbers, *r* = -0.01, *p* = 0.945, 95% CI [-0.20,0.19].

#### Sex Differences

We next examined whether participant sex moderated the way numbers are gendered. First, we examined whether participant sex moderated IAT scores. This analysis excludes 15 participants who failed to enter their identification code when completing the IAT (thus making it impossible to link their IAT score back to their demographic information) and three additional participants who did not indicate their sex. This leaves a total of 66 women and 35 men in the analysis. A large sex difference emerged between the IAT *D*-scores of men [*M* = -0.14, SD = 0.55, 95% CI (-0.32,0.05)] vs. women (*M* = 0.43, SD = 0.44, 95% CI [0.32,0.53]), *t*(99) = 5.63, *p* < 0.001, Hedges’s *g*_s_ = 1.17. While the mean *D*-score of women was significantly different from 0, *t*(65) = 7.85, *p* < 0.001, the mean *D*-score for men was not, *t*(34) = 1.51, *p* = 0.14. Thus, at an implicit level, the gendering of numbers was moderately strong for women but absent for men, on average.

To examine potential sex differences in the explicit gendering of numbers, we compared the overall explicit gender contrast scores (described in the preceding section; positive scores reflect a greater tendency to associate masculinity with odd integers and femininity with even integers) as a function of participant sex. In terms of the masculinity/femininity contrast, the difference between men and women was marginally significant, *t*(112) = 1.95, *p* = 0.054, Hedges’s *g*_s_ = 0.38. While women showed a marginally stronger numerical gendering pattern than men [*M* (SD) = 1.37 (1.64) vs. 0.76 (1.47), respectively], both of these means were significantly greater than 0, reflecting the expected pattern of numerical gender associations; for men, *t*(38) = 3.22, *p* = 0.003; for women, *t*(74) = 7.24, *p* < 0.001. Results of analyses of the agency/communion composite were very similar; in this case, the difference between men and women was significant, *t*(112) = 2.32, *p* = 0.022, Hedges’s *g*_s_ = 0.45. Women showed a stronger numerical gendering pattern than men [*M* (SD) = 1.34 (1.53) vs. 0.67 (1.32), respectively], but again, both of these means were significantly greater than 0, reflecting the expected pattern of numerical gender associations; for men, *t*(38) = 3.19, *p* = 0.003, and for women, *t*(74) = 7.58, *p* < 0.001. Thus, when it comes to explicit perceptions of the single-digit integers, both men and women exhibit numerical gendering, but the pattern was more pronounced among women, with a moderately sized sex difference.

In terms of evaluative reactions to numbers, men and women did not differ in their composite liking of odd integers [*M* (SD) = 4.52 (0.84) vs. 4.50 (0.86), respectively], *t*(112) = 0.14, *p* = 0.89, Hedges’s *g*_s_ = 0.03, but women exhibited greater liking of the even integers than men did [*M* (SD) = 4.96 (0.82) vs. 4.41 (0.67), respectively], *t*(112) = 3.58, *p* = 0.001, Hedges’s *g*_s_ = 0.70. Men did not show reliably differential liking of 1-digit numbers as a function of their odd vs. even status, *t*(38) = 0.91, *p* = 0.369, Hedges’s *g*_rm_ = 0.14, but women liked even numbers better than odd ones, *t*(74) = 3.56, *p* = 0.001, Hedges’s *g*_rm_ = 0.55.

### Discussion

Overall, these results converge in a consistent way on the conclusion that even numbers seem feminine and odd numbers seem masculine. At the explicit level, this was true for both direct ratings of gender as well as for ratings of gender-associated traits reflecting agency and communion. These explicit biases were evident in both men and women, but the effects were larger for women than men. The tendency to automatically associate numbers with gender based on their even/odd status in an IAT was reliably found among women, but not among men. Collectively, these findings point to the conclusion that odd vs. even numbers are differentially gendered, especially for women.

Assessment of the liking of single-digit integers suggests that even numbers seem nicer, but evaluations of these numbers is complicated by the fact that these frequently encountered numerals are often imbued with special personal or cultural significance. For example, numbers are differentially liked depending on whether they are considered lucky (e.g., Jiang et al., 2009), are associated with one’s birth month/day (Kitayama and Rarasawa, 1997), are associated with holidays, have religious associations, or are linked to other culturally prominent numerical practices (e.g., Stieger and Krizan, 2013). Such associations are certainly likely to influence affective reactions to numbers. In the next study, we attempted to replicate these initial findings using a methodology that greatly dilutes these kinds of systematic personal and cultural associations.

## Study 2

If odd/even status has a general association with masculinity/femininity, this pattern should be evident not only with the single-digit integers but also with larger numbers. In the second experiment we examined 2-digit numbers. In addition to replicating the basic findings of the first experiment, an examination of 2-digit numbers allowed us to address additional theoretical issues. First, we examined whether the gendering of 2-digit numbers depends solely on the final digit (i.e., on whether the number *as a whole* is even or odd), or if instead it might depend on an additive process wherein the overall gender connotations of the number are influenced by both digits. If so, then an odd number composed of 2 odd digits (e.g., 73) might be perceived as more masculine than an odd number with just 1 final odd digit (e.g., 83). Conversely, an even number composed of two even digits (e.g., 42) might seem more feminine than one with just 1 final even digit (e.g., 52). Second, we examined the pattern of greater liking for even than odd numbers in a context where other numerical associations (e.g., luckiness) are minimized. This would be hard to accomplish in the case of the single-digit numbers, but in the second experiment, we used randomly selected samples of odd and even 2-digit numbers, such that any systematic personal or cultural associations bearing on the likableness of the specific rated numerical stimuli would be highly unlikely.

### Method

#### Participants and Design

We recruited a sample of 196 Americans who from Amazon Mechanical Turk, who participated in a computerized survey experiment in return for a payment of $0.75. The sample consisted of 113 women, 81 men, and 2 unspecified gender, was 84.2% White, and ranged in age from 19 to 74 (*M* = 35.1, SD = 12.82). The research was approved by the Institutional Review Board of Northwestern University, and informed consent was obtained from all participants.

#### Stimuli and Procedure

Participants were instructed that the experiment would examine the reactions that people have to various numbers, given the ubiquity of encountering numbers in daily life. They then each rated 32 different 2-digit numbers in terms of how masculine, feminine, and likable they were, in each case on a 7-point scale from 1 (“not at all”) to 7 (“extremely”). The numbers that participants rated were randomly selected subsets of four kinds of 2-digit numbers: those that consist of two odd individual digits (e.g., 35, 93), those that consist of two even digits (e.g., 46, 82), those that have an even first digit and an odd second digit (e.g., 21, 65), and those that have an odd first digit and an even second digit (e.g., 34, 58); participants rated eight randomly selected examples of each type of number; as each number appeared, participants made the three ratings (masculinity, femininity, likableness) before moving on to the next number.

### Results and Discussion

#### Gender Perceptions

**FIGURE 2:**
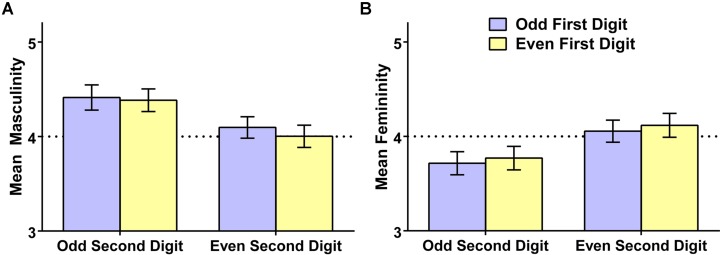
**Mean masculinity ratings **(A)** and femininity ratings **(B)**, and 95% confidence intervals, of 2-digit numbers as a function of whether the first and second digit are even or odd, Study 2**. The dotted line reflects the rating scale midpoint.

For each participant, we computed average ratings of the eight randomly selected exemplars they evaluated from each of the four possible number types. We then conducted a repeated-measures analysis of variance examining these mean ratings as a function of whether the first digit was even or odd and whether the second digit was even or odd. Results, presented in **Figure 2**, were clear. With respect to perceived masculinity [panel (A)], there was no difference as a function of whether the first digit was odd or even, *M* (SD) = 4.25 (0.64) vs. 4.19 (0.58), *t*(193) = 1.42, *p* = 0.16, Hedges’s *g*_rm_ = 0.10; however, there was a moderate-sized effect of the second digit, with odd numbers resulting in significantly greater perceived masculinity than even numbers, *M* (SD) = 4.40 (0.80) vs. 4.05 (0.73), *t*(193) = 4.39, *p* < 0.001, Hedges’s *g*_rm_ = 0.46. There was no interactive effect of first and second digit, *F* < 1. With respect to perceived femininity [panel (B)], there was again no effect of the first digit, *M* (SD) = 3.89 (0.61) vs. 3.94 (0.63) for odd vs. even, respectively, *t*(193) = 1.40, *p* = 0.16, Hedges’s *g*_rm_ = 0.09; however, there was a significant effect of the second digit, with even numbers resulting in greater perceived femininity than odd numbers, *M* (SD) = 4.09 (0.78) vs. 3.74 (0.79), *t*(193) = 4.29, *p* < 0.001, Hedges’s *g*_rm_ = 0.44. There was again no interactive effect of first and second digit, *F* < 1. The figure also makes clear that the overall gendering effect is localized in the odd numbers. Numbers that were odd (based on their second digit) were perceived to be more masculine than feminine [*M* (SD) = 4.40 (0.80) vs. 3.74 (0.79)], *t*(193) = 7.00, *p* < 0.001, Hedges’s *g*_rm_ = 0.82, but numbers that were even (based on their second digit) were not perceived to be more feminine than masculine [*M* (SD) = 4.05 (0.73) vs. 4.09 (0.78)], *t*(193) = 0.41, *p* = 0.678, Hedges’s *g*_rm_ = 0.05. Thus, the gender of 2-digit numbers was determined by whether the number as a whole was odd or even (i.e., by the right-most digit); the first digit exerted no detectable influence on gender perceptions. This pattern accords with evidence that people access their stored semantic representations of Arabic numbers on the basis of the rightmost digit (Dehaene et al., 1993). Overall, odd 2-digit numbers elicited a large gendering effect, being rated as relatively high in masculinity and relatively low in femininity, whereas even 2-digit numbers were not seen as particularly masculine or feminine in the sample as a whole.

#### Liking

**FIGURE 3:**
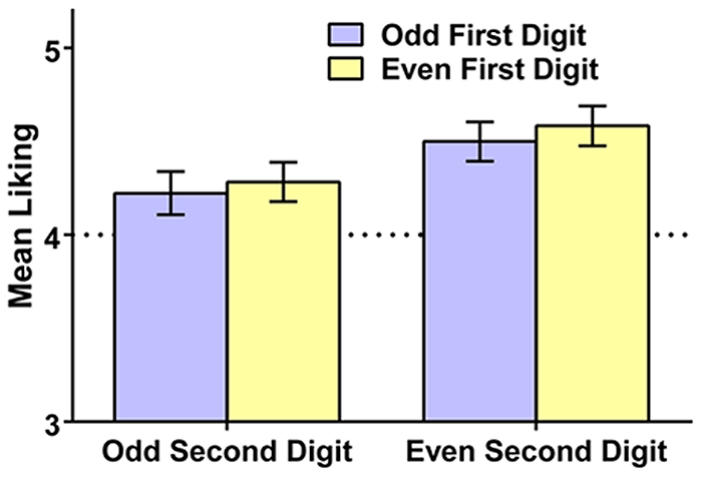
**Mean liking of 2-digit numbers (and 95% confidence intervals) as a function of whether the first and second digit are even or odd, Study 2**. The dotted line reflects the rating scale midpoint.

We next examined whether liking for the 2-digit numbers was influenced by their odd/even status. We found significant effects of both the first and second digit on liking (see **Figure 3**). Respondents liked numbers with an even first digit more than numbers with an odd first digit [*M* (SD) = 4.43 (0.59) vs. 4.36 (0.62)], *t*(193) = 2.23, *p* = 0.027, Hedges’s *g*_rm_ = 0.12. Respondents also liked numbers with an even second digit more than numbers with an odd second digit [*M* (SD) = 4.54 (0.68) vs. 4.25 (0.71)], *t*(193) = 4.94, *p* < 0.001, Hedges’s *g*_rm_ = 0.41. There was no interaction of first and second digit, *F* < 1. Thus, there was a general tendency for people to like 2-digit numbers containing even digits more than ones containing odd digits. Though the first digit was able to contribute to this form of bias, the second digit had a markedly larger effect on liking, suggesting that the number’s overall status as even versus odd was the primary driver of liking.

#### Sex Differences

**FIGURE 4:**
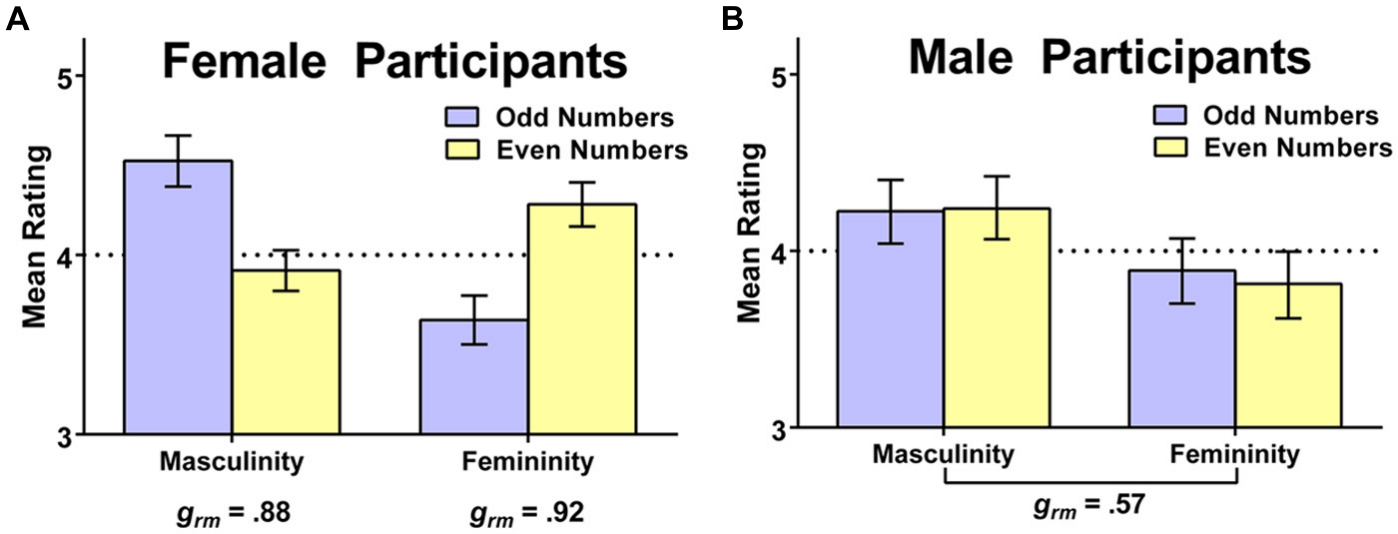
**Visual summary of sex-specific patterns in the gendering of 2-digit numbers (Study 2)**. Women **(A)** ascribed gender based on whether numbers were odd or even, while men **(B)** viewed all numbers as more masculine than feminine; standardized effect sizes (Hedges’s *g*_rm_) appear below the relevant comparison and the dotted line reflects the rating scale midpoint.

We again examined whether participant sex would moderate the obtained results. We observed clear moderating effects of participant sex in gendered perceptions of double-digit numbers. With respect to perceived masculinity, participant sex moderated the effect of an odd vs. even second digit, *F*(1,192) = 16.33, *p* < 0.001, $\eta_{p}^{2}$ = 0.08; female participants viewed odd numbers as more masculine than even numbers, *M* (SD) = 4.52 (0.77) vs. 3.91 (0.61), *t*(113) = 5.86, *p* < 0.001, but male participants did not, *M* (SD) = 4.22 (0.82) vs. 4.24 (0.83), *t*(80) = 0.14, *p* = 0.89; instead, the men rated both types of numbers as relatively masculine. With respect to perceived femininity, sex again moderated the effect of odd vs. even second digit, *F*(1,192) = 21.91, *p* < 0.001, $\eta_{p}^{2}$ = 0.10; female participants viewed even numbers as more feminine than odd numbers, *M* (SD) = 4.28 (0.66) vs. 3.64 (0.73), *t*(112) = 6.38, *p* < 0.001, but male participants did not, *M* (SD) = 3.81 (0.86) vs. 3.89 (0.84), *t*(80) = 0.67. Instead, men tended to rate the numbers as relatively low in femininity irrespective of odd/even status. These sex-specific patterns of numerical gendering are summarized in **Figure 4**. Results for women, shown in panel **(A)**, reveal a large effect of even/odd status on ascribed numerical gender, whereas results for men, summarized in panel **(B)**, show that men generally rated all the numbers as more masculine than feminine. Thus, overall, men and women agreed in viewing odd 2-digit numbers as relatively more masculine than feminine, but they disagreed in their view of even 2-digit numbers; women perceived them to be more feminine than masculine, but men again viewed them as more masculine than feminine. This general tendency for men to view all of these larger (i.e., 2-digit) numbers as generally masculine might result if men view mathematics (and, hence, numbers) as being a masculine domain.

Unlike Study 1, we found no participant sex effect (*F* < 1) on liking for 2-digit numbers as a function of whether they were even or odd. The tendency to like even numbers more than odd ones was shared by both sexes.

## General Discussion

Numbers can evoke diverse feelings and associations (Bellos, 2014). The present experiments provided consistent, clear-cut, and direct evidence that numbers are gendered, but in different ways and to different degrees for women and men. Comparisons between even and odd numbers consistently revealed that odd numbers seem more masculine than feminine, but the tendency to see even numbers as more feminine than masculine was only consistently found among women. For men, single-digit even numbers seemed more feminine than masculine, but when it came to 2-digit numbers, men regarded them to be *generally* more masculine than feminine, regardless of whether they were even or odd. This finding helps explain the gender differences we observed on the IAT. To the extent that men generally view all larger numbers as masculine, they would not be expected to show any IAT effect differentially associating number parity with masculinity vs. femininity.

Because men and women converge in their tendency to see odd numbers as more masculine than feminine, the even/odd numerical gendering effect was more pronounced for odd than for even numbers. Looking at the gender ratings of individual 1-digit numbers (**Table 1**) reveals that the observed gender-connotation effects were larger for odd numbers than for even numbers. Among the 2-digit numbers, differential perceptions of masculine vs. feminine qualities in the sample as a whole were found only for odd numbers. These results place noteworthy constraints on the phenomenon of numerical gender.

The possibility that liking for numbers would be affected by their parity status was evident among both single-digit and double-digit numbers. Moreover, both the first and the second digit contributed to the effect of odd/even status on liking for the 2-digit numbers. In Study 1, women liked even 1-digit numbers to a greater degree than men, while both liked odd numbers to a similar extent. In the case of 2-digit numbers, men and women both preferred even numbers to odd ones.

Numbers play an important role in our lives, and how we respond to numbers can have a noteworthy influence on personal decisions. The present findings lead to a range of interesting hypotheses for future investigation. For example, it is well known that consumers are sensitive to the gender connotations of potential purchases (e.g., Fugate and Phillips, 2010) and gendered features such as a product’s shape or color can influence consumer preferences (e.g., Funk and Ndubisi, 2006). The present findings suggest that numbers associated with a product (e.g., its price) might influence its perceived gender appropriateness in subtle ways (see Wilkie and Bodenhausen, 2012). Such an effect would be most likely to emerge under conditions where the gender status of the product is ambiguous (e.g., unisex products). Numbers are also important in educational and career contexts, particularly in regard to science, technology, engineering, and mathematics (STEM) fields, which all have numerical manipulations (e.g., counting, measuring, etc.) as their fundamental basis. Women have long been underrepresented in these fields, and undoubtedly a variety of factors contribute to this gender disparity. Women have been shown to perform worse on mathematical tests when gender identity is salient, whereas men’s math performance can enjoy a boost when gender is salient (e.g., Schmader, 2002; Walton and Cohen, 2003). Our finding that larger (i.e., 2-digit) numbers were seen by men as being generally more masculine than feminine may imply that men commonly experience an alignment between numbers and their (typically) masculine identities, and this may help them feel more comfortable with and entitled to engage with numbers than women do in educational and vocational contexts. Prior research has documented that, compared to females, males tend to have higher levels of interest in math (e.g., Su et al., 2009) and more positive attitudes toward math (Else-Quest et al., 2010). Similarly, male students tend to have stronger linkages between mathematics and their self-concepts than female students do (Skaalvik and Skaalvik, 2004). These differences matter, in that they can influence educational aspirations and choices, such as whether or not to take advanced math courses (Chipman et al., 1985; Köller et al., 2001). An interesting question for future research concerns the extent to which intuitions about numerical gender are related to these patterns of sex differences in math interest, attitudes, and identification. Much research attests to the fact that feeling a sense of personal fit with one’s surrounding environment is important in educational and vocational contexts (e.g., Kulik et al., 1987). If men perceive that numbers are masculine, then they may feel a greater sense of personal fit with math-oriented settings. Conversely, if women feel that numbers—particularly odd ones—are not congruent with a feminine identity, then their sense of fit may be undermined. Indeed, Diekman et al. (2011) argued that STEM careers often seem more appealing to women when they are perceived to align with their communal goals and identities. More research will be needed in order to determine whether and how numerical gendering is related to the pattern of sex differences that have been documented in the area of mathematical interests and attitudes.

The present findings emerged from a sample of (primarily White) American adults; thus, one limitation of these studies is that they cannot speak to the generalizability of the findings across different cultural populations or to children. Wilkie and Bodenhausen (2012) provided tentative evidence that South Asians perceive the concept of “odd numbers” to be more masculine than the concept of “even numbers,” but more research is needed to determine how universal vs. culture-specific the gendering of numbers might be.

Overall, the present results point to the potency of gender as a reference point for understanding and conceptualizing the world around us. Even something as basic and abstract as number parity can carry connotations of gender. Gender research has often been marginalized and treated as a specialty niche of lesser importance (e.g., Eagly et al., 2012), but gender is fundamental to the human mind. The present findings underscore the pervasiveness of gender in our lives. Even our numbers are gendered.

## Conflict of Interest Statement

The authors declare that the research was conducted in the absence of any commercial or financial relationships that could be construed as a potential conflict of interest.
